# Crystal structure and Hirshfeld surface analysis of bis­(2,6-di­amino­pyridinium) tetra­chlorido­cobaltate(II)

**DOI:** 10.1107/S2056989018003171

**Published:** 2018-03-02

**Authors:** Oumaima Ben Moussa, Hammouda Chebbi, Mohamed Faouzi Zid

**Affiliations:** a University of Tunis El Manar, Faculty of Sciences of Tunis, Laboratory of Materials, Crystal Chemistry and Applied Thermodynamics, 2092 El Manar II, Tunis, Tunisia; b University of Tunis, Preparatory Institute for Engineering Studies of Tunis, Street Jawaher Lel Nehru, 1089 Montfleury, Tunis, Tunisia

**Keywords:** cobalt(II), hybrid organic–inorganic materials, crystal structure, Hirshfeld surface, fingerprint plots

## Abstract

In the title mol­ecular salt, (C_5_H_8_N_3_)_2_[CoCl_4_], the cations are protonated at their pyridine N atoms and the anion is an almost regular tetra­hedron. The crystal structure consists of alternating inorganic layers, built from tetra­chlorido­cobaltate anions, and organic layers formed by protonated cations of 2,6-di­amino­pyridinium. The crystal packing is governed by C/N—H⋯Cl hydrogen-bonding inter­actions between the organic and the inorganic ions and Cl⋯Cl inter­actions. Moreover, the cations show a π–π stacking inter­action [inter­centroid distance = 3.763 (2) Å]. The prevalence of these inter­actions is illustrated by an analysis of the three-dimensional Hirshfeld surface and by two-dimensional fingerprint plots.

## Abstract 2

The crystal structure of the title mol­ecular salt features N—H⋯Cl and C—H⋯Cl hydrogen bonds and π–π inter­actions; Hirshfeld surface analysis and fingerprint plots are reported.

## Chemical context   

One of the best studied groups of organic–inorganic hybrid materials are the cobalt(II) halide compounds because of their important properties such as fluorescence and magnetism (Decaroli *et al.*, 2015 ▸; Kurmoo, 2009 ▸). The coordination sphere of Co^II^ is variable, leading to different geometries including octa­hedral, tetra­hedral, square pyramidal, trigonal bipyramidal and square planar (Kurmoo, 2009 ▸). Pyridine as an organic heterocyclic mol­ecule has various biological activities (Sellin, 1981 ▸; Davidson *et al.*, 1988 ▸). As part of our studies in this area, the title compound, (C_5_H_8_N_3_)_2_[CoCl_4_] (I), has been investigated.
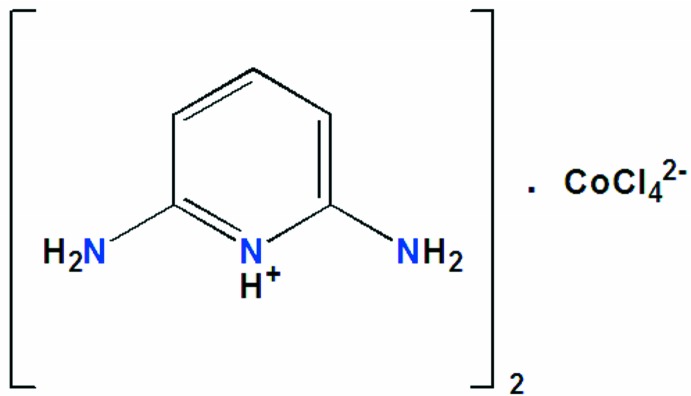


## Structural commentary   

**Figure 1:**
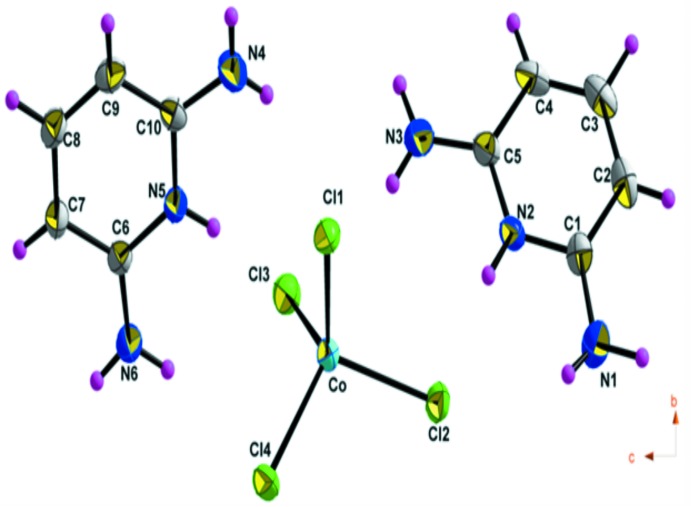
The asymmetric unit of (I), with displacement ellipsoids are drawn at the 30% probability level.

**Figure 2:**
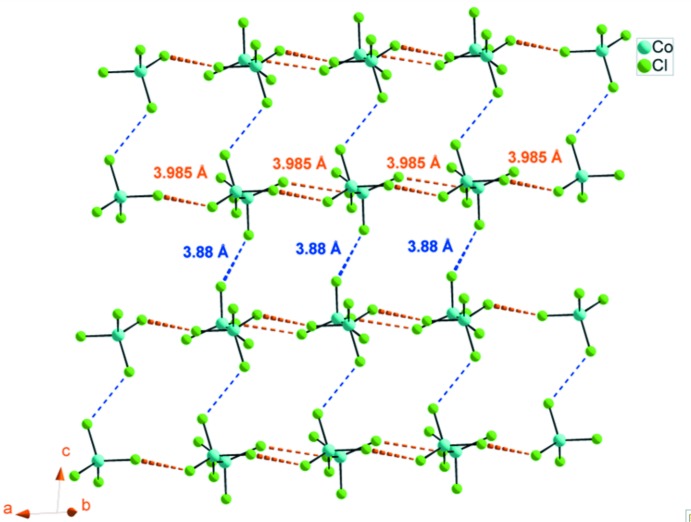
The inter­anionic Cl⋯Cl contact in the tetra­chloro­cobaltate anion of (I) along the *a* and *c* axis.

The asymmetric unit of (I) is made up of one tetra­chlorido­cobaltate, [CoCl_4_]^2−^, anion and two protonated 2,6-di­amino­pyridinium, (C_5_H_8_N_3_)^+^, organic cations (Fig. 1 ▸). The geometry of the CoCl_4_ anion is characterized by a range of Co—Cl bond length from 2.2595 (14) to 2.2795 (13) Å and Cl—Co—Cl angles varying from 106.44 (5) to 112.69 (5)°, building a slightly distorted tetra­hedron. These data are in agreement with those found in related compounds (Mghandef & Boughzala, 2015 ▸). The calculated average values of the distortion indice as described by Baur (1974 ▸) corresponding to the different lengths and angles in the CoCl_4_ tetra­hedra [ΔI(Co—Cl) = 0.004 and ΔI(Cl—Co—Cl) = 0.0019] show a slight distortion of the tetra­hedra. The inter­anionic Cl⋯Cl contact distances between the nearest neighbor tetra­hedra are 3.986 (2) Å along the *a* axis and 3.889 (2) Å along the *c* axis (Fig. 2 ▸), compared to a van der Waals contact distance of 3.50 Å. These contacts are sometimes associated with weak anti­ferromagnetic inter­actions (Shapiro *et al.*, 2007 ▸), which decrease rapidly with increasing Cl⋯Cl separation.

Pyridinium cations always possess an expanded angle of C—N—C in comparison with the parent pyridine (Ben Nasr *et al.*, 2015 ▸). Thus, the observed angles in (I) of C1—N2—C5 and C6—N5—C10 are 124.2 (3) and 124.1 (3)°, respectively, are wider than that in neutral pyridine (116.6°), indicating that protonation takes place on the pyridine ring N2 and N5 atoms. Accordingly, within the cations, we note that the N—C and C—C distances range from 1.332 (5) to 1.393 (6) Å, while the C—C—C, N—C—N, C—C—N and N—C—C angles vary from 116.00 (4) to 126.50 (4)°. The 2,6-di­amino­pyridinium units are essentially planar, with an r.m.s. deviation from the mean plane of 0.002 and 0.006 Å for the N2 and N5 species, respectively.

## Supra­molecular features   

**Figure 3:**
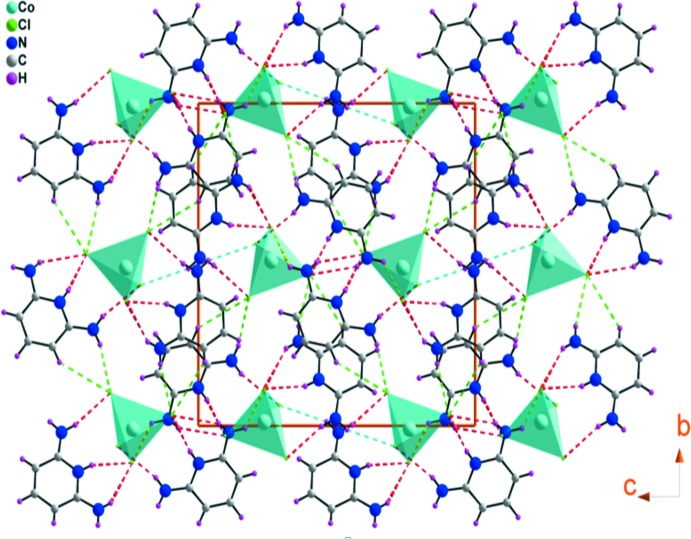
View of (I) towards the *bc* plane. The dotted lines indicate hydrogen bonds.

Examination of the crystal structure of (I) reveals organic layers parallel to the *a* *b* plane made of 2,6-di­amino­pyridinium cations alternating with inorganic layers formed by tetra­chlorido­cobaltate anions (Fig. 3 ▸), which is similar to those of related materials: (C_5_H_6_Br_2_N_3_)_2_[*M*Br_4_] (*M* = Cd, Mn) (Al-Far *et al.*, 2009 ▸) and (C_5_H_7_N_2_)_2_[CoBr_4_] (Mhadhbi *et al.*, 2016 ▸).

**Figure 4:**
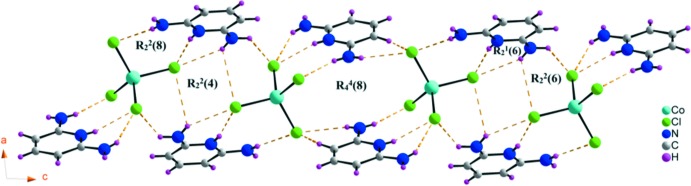
Projection of a part of the crystalline structure of the compound (I), showing the formation of the motifs 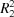
(4), 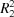
(6), 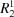
(6), 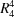
(8) and 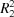
(8).

The construction of the three-dimensional architecture is consolidated by N—H⋯Cl and C—H⋯Cl hydrogen bonds (Table 1 ▸), generating 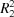
(4), 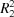
(6), 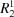
(6), 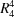
(8) and 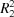
(8) graph-set motifs (Fig. 4 ▸).

**Figure 5:**
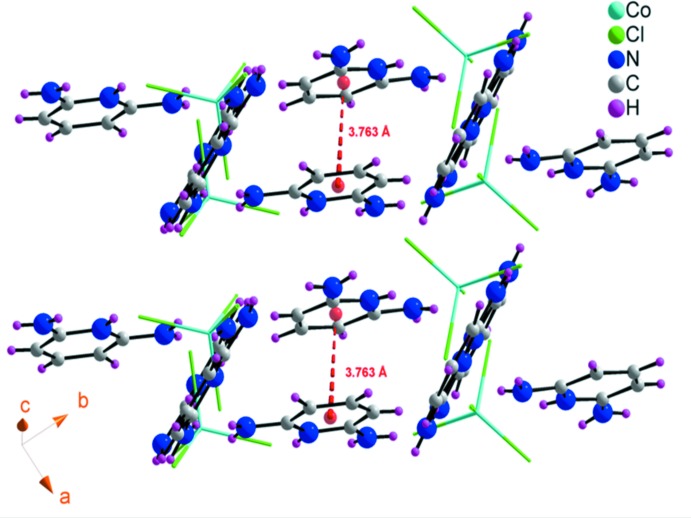
π–π stacking inter­actions between the neighboring aromatic organic cations in (I). The inorganic anions are shown as sticks for clarity.

As can be seen from Fig. 5 ▸, the two nearest neighboring anti-parallel organic cations, which are not connected by hydrogen bonding, are stacked in a face-to-face mode. The centroid–centroid distance is 3.762 (5) Å, slightly less than 3.8 Å, which is the maximum value accepted for π–π inter­actions (Ben Hassen *et al.*, 2017 ▸; Janiak, 2000 ▸).

## Hirshfeld surface analysis   

**Figure 6:**
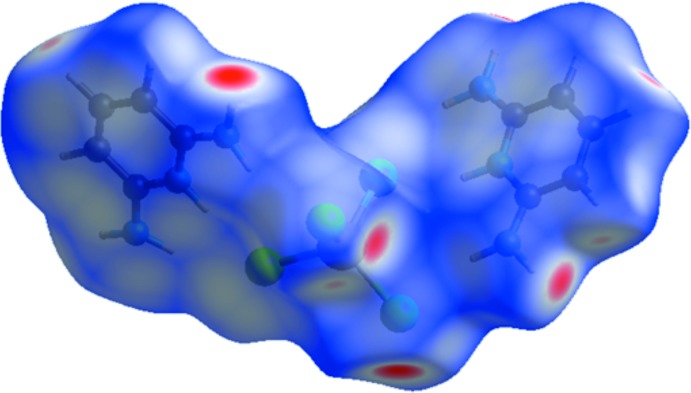
View of the Hirshfeld surface of (I) mapped over *d*_norm_.

**Figure 7:**
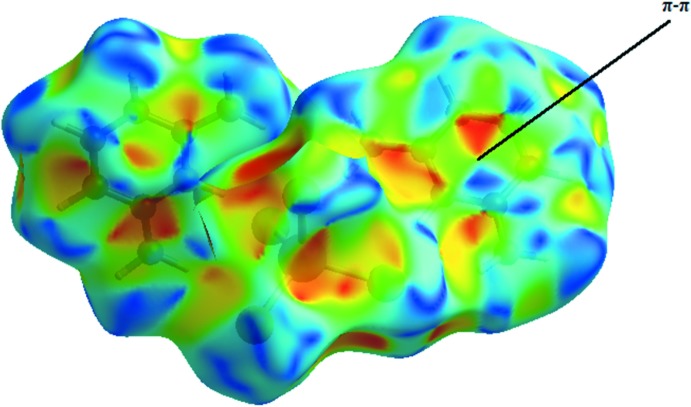
Hirshfeld surface mapped over shape-index, highlighting the regions involved in π–π stacking inter­actions.

The Hirshfeld surface (Spackman & Jayatilaka, 2009 ▸) and the associated two-dimensional fingerprint plots were performed with *CrystalExplorer* (Wolff *et al.*, 2012 ▸). The Hirshfeld surface of the title compound mapped over *d*_norm_ is illustrated in Fig. 6 ▸. The red spots correspond to the H⋯Cl close contacts, which are due to the N—H⋯Cl hydrogen bonds. Similarly, the presence of H⋯Cl contacts (due to C—H⋯Cl hydrogen bonds) are indicated by a light-red color. The white areas correspond to the places where the distance separating neighboring atoms are close to the sum of the van der Waals radius of the considered atoms and indicate H⋯H inter­actions. The bluish areas illustrate areas where neighboring atoms are too far apart for there to be inter­actions between them. In the shape-index map (Fig. 7 ▸), the adjacent red and blue triangle-like patches show concave regions that indicate π–π stacking inter­actions (Bitzer *et al.*, 2017 ▸).

**Figure 8:**
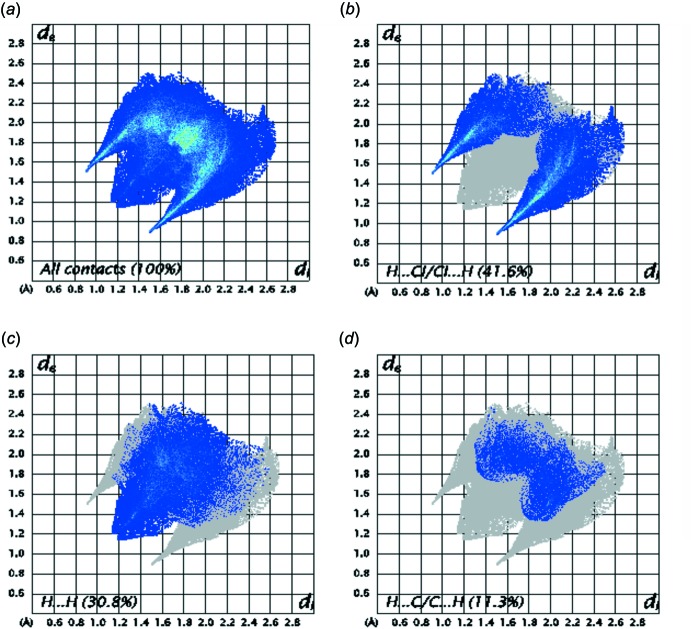
The two-dimensional fingerprint plots of (I), (*a*) showing all inter­actions and delineated into H⋯Cl/Cl⋯H (*b*), H⋯H (*c*) and C⋯H/H⋯C (*d*) inter­actions.

The fingerprint plots of (I) (Fig. 8 ▸ *a*) (Parkin *et al.*, 2007 ▸; Rohl *et al.*, 2008 ▸), reveal that the main inter­molecular inter­actions with the highest percentage contributions are: H⋯Cl/Cl⋯H (41.6%, Fig. 8 ▸ *b*), H⋯H (30.8%, Fig. 8 ▸ *c*) and C⋯H/H⋯C (11.3%, Fig. 8 ▸ *d*).

**Figure 9:**
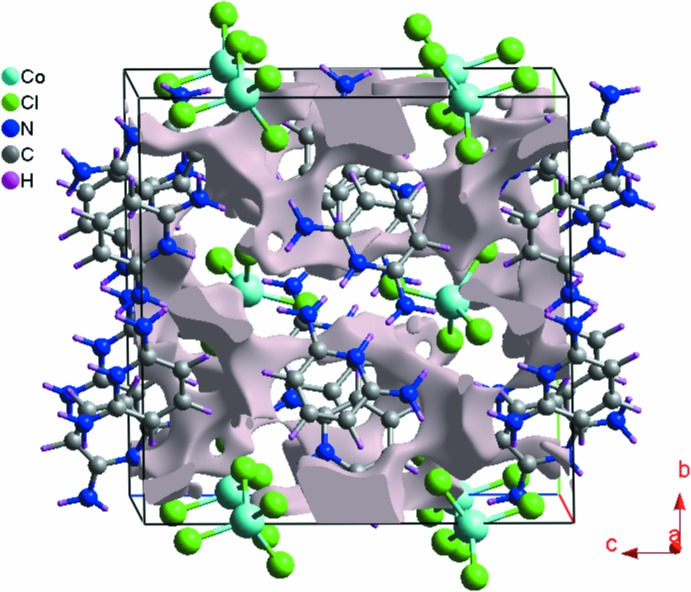
Void plot for (I).

Fig. 9 ▸ shows the voids (Wolff *et al.*, 2012 ▸) in the crystal structure of (I). These are based on the sum of spherical atomic electron densities at the appropriate nuclear positions (procrystal electron density). The crystal voids calculation (results under 0.002 a.u. isovalue) shows the void volume of title compound to be of the order of 172 Å^3^ and surface area in the order of 648 Å^2^. With the porosity, the calculated void volume of (I) is 10%. There are no large cavities. We note that the electron-density isosurfaces are not completely closed around the components, but are open at those locations where inter­species approaches are found, *e.g*. N—H⋯Cl and C—H⋯Cl.

## Synthesis and crystallization   

2,6-di­amino­pyridine and CoCl_2_·6H_2_O (molar ratio 1:1) were dissolved in 10 ml of methanol; 3 ml of hydro­chloric acid (37%) was added dropwise to the mixture and the resulting blue solution was put aside for crystallization at room temperature. After two weeks, blue crystals of (I) were recovered.

## Refinement   

Crystal data, data collection and structure refinement details are summarized in Table 2 ▸. All hydrogen atoms were found in a difference-Fourier map and refined isotropically.

## Supplementary Material

Crystal structure: contains datablock(s) I. DOI: 10.1107/S2056989018003171/hb7732sup1.cif

Structure factors: contains datablock(s) I. DOI: 10.1107/S2056989018003171/hb7732Isup2.hkl

CCDC reference: 1588020

Additional supporting information:  crystallographic information; 3D view; checkCIF report

## Figures and Tables

**Table 1:** Hydrogen-bond geometry (Å, °)

*D*—H⋯*A*	*D*—H	H⋯*A*	*D*⋯*A*	*D*—H⋯*A*
N1—H2*N*1⋯Cl2	0.84 (5)	2.69 (5)	3.432 (4)	147 (4)
N4—H2*N*4⋯Cl2^i^	0.84 (5)	2.65 (5)	3.465 (5)	162 (4)
N6—H1*N*6⋯Cl4	0.85 (5)	2.58 (5)	3.406 (4)	165 (4)
N2—H1*N*2⋯Cl2	0.82 (4)	2.44 (4)	3.240 (3)	168 (4)
N5—H1*N*5⋯Cl1	0.82 (5)	2.43 (5)	3.191 (3)	156 (4)
N3—H1*N*3⋯Cl4^ii^	0.88 (4)	2.53 (5)	3.390 (5)	166 (4)
N4—H1*N*4⋯Cl1	0.79 (4)	2.79 (4)	3.481 (5)	147 (4)
N6—H2*N*6⋯Cl3^iii^	0.82 (5)	2.75 (5)	3.516 (4)	156 (4)
N3—H2*N*3⋯Cl3	0.83 (6)	2.61 (6)	3.420 (5)	166 (6)
N1—H1*N*1⋯Cl1^iv^	0.94 (5)	2.69 (5)	3.326 (4)	126 (4)
C7—H7⋯Cl3^iii^	0.91 (4)	2.89 (4)	3.669 (4)	145 (3)

Symmetry codes: (i) 
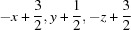
; (ii) 
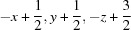
; (iii) 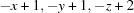; (iv) 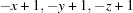.

**Table 2:** Experimental details

Crystal data	
Chemical formula	(C_5_H_8_N_3_)_2_[CoCl_4_]
*M*_r_	421.02
Crystal system, space group	Monoclinic, *P*2_1_/*n*
Temperature (K)	293
*a*, *b*, *c* (Å)	7.390 (4), 15.373 (4), 15.387 (4)
β (°)	98.203 (4)
*V* (Å^3^)	1730.1 (11)
*Z*	4
Radiation type	Mo *K*α
μ (mm^−1^)	1.61
Crystal size (mm)	0.4 × 0.3 × 0.1

Data collection	
Diffractometer	Enraf–Nonius CAD-4
Absorption correction	ψ scan (North *et al.*, 1968 ▸)
*T*_min_, *T*_max_	0.777, 0.998
No. of measured, independent and observed [*I* > 2σ(*I*)] reflections	4367, 3770, 2396
*R*_int_	0.038
(sin θ/λ)_max_ (Å^−1^)	0.638

Refinement	
*R*[*F* ^2^ > 2σ(*F* ^2^)], *wR*(*F* ^2^), *S*	0.044, 0.110, 1.01
No. of reflections	3770
No. of parameters	255
H-atom treatment	All H-atom parameters refined
Δρ_max_, Δρ_min_ (e Å^−3^)	0.37, −0.42

Computer programs: *CAD-4 EXPRESS* (Duisenberg, 1992 ▸; Macíček & Yordanov, 1992 ▸), *XCAD4* (Harms & Wocadlo, 1995 ▸), *SHELXS97* (Sheldrick, 2008 ▸), *SHELXL2014* (Sheldrick, 2015 ▸), *DIAMOND* (Brandenburg, 2006 ▸), *WinGX* (Farrugia, 2012 ▸) and *publCIF* (Westrip, 2010 ▸).

## supplementary crystallographic information

### Crystal data

**Table:**

(C_5_H_8_N_3_)_2_[CoCl_4_]	*F*(000) = 852
*M**_r_* = 421.02	*D*_x_ = 1.616 Mg m^−^^3^*D*_m_ = 1.616 Mg m^−^^3^*D*_m_ measured by ?
Monoclinic, *P*2_1_/*n*	Mo *K*α radiation, λ = 0.71073 Å
*a* = 7.390 (4) Å	Cell parameters from 25 reflections
*b* = 15.373 (4) Å	θ = 10.1–14.9°
*c* = 15.387 (4) Å	µ = 1.61 mm^−^^1^
β = 98.203 (4)°	*T* = 293 K
*V* = 1730.1 (11) Å^3^	Parallelepiped, blue
*Z* = 4	0.4 × 0.3 × 0.1 mm

### Data collection

**Table:**

Enraf–Nonius CAD-4 diffractometer	2396 reflections with *I* > 2σ(*I*)
Radiation source: fine-focus sealed tube	*R*_int_ = 0.038
Graphite monochromator	θ_max_ = 27.0°, θ_min_ = 2.7°
ω/2θ scans	*h* = −9→1
Absorption correction: ψ scan (North *et al.*, 1968)	*k* = −1→19
*T*_min_ = 0.777, *T*_max_ = 0.998	*l* = −19→19
4367 measured reflections	2 standard reflections every 120 reflections
3770 independent reflections	intensity decay: 1%

### Refinement

**Table:**

Refinement on *F*^2^	Hydrogen site location: difference Fourier map
Least-squares matrix: full	All H-atom parameters refined
*R*[*F*^2^ > 2σ(*F*^2^)] = 0.044	*w* = 1/[σ^2^(*F*_o_^2^) + (0.0522*P*)^2^] where *P* = (*F*_o_^2^ + 2*F*_c_^2^)/3
*wR*(*F*^2^) = 0.110	(Δ/σ)_max_ = 0.001
*S* = 1.01	Δρ_max_ = 0.37 e Å^−^^3^
3770 reflections	Δρ_min_ = −0.42 e Å^−^^3^
255 parameters	Extinction correction: SHELXL2014 (Sheldrick, 2015), Fc^*^=kFc[1+0.001xFc^2^λ^3^/sin(2θ)]^-1/4^
0 restraints	Extinction coefficient: 0.0049 (8)

### Special details

**Table:**

Geometry. All esds (except the esd in the dihedral angle between two l.s. planes) are estimated using the full covariance matrix. The cell esds are taken into account individually in the estimation of esds in distances, angles and torsion angles; correlations between esds in cell parameters are only used when they are defined by crystal symmetry. An approximate (isotropic) treatment of cell esds is used for estimating esds involving l.s. planes.

### Fractional atomic coordinates and isotropic or equivalent isotropic displacement parameters (Å^2^)

**Table:**

	*x*	*y*	*z*	*U*_iso_*/*U*_eq_	
Co1	0.44824 (7)	0.51081 (3)	0.73764 (3)	0.03878 (16)	
Cl1	0.66787 (14)	0.61509 (6)	0.73884 (6)	0.0497 (2)	
Cl2	0.36703 (14)	0.46854 (6)	0.59572 (6)	0.0500 (3)	
Cl3	0.20113 (14)	0.56398 (7)	0.79102 (6)	0.0547 (3)	
Cl4	0.58007 (17)	0.39816 (6)	0.81784 (7)	0.0627 (3)	
N2	0.1002 (5)	0.6175 (2)	0.4994 (2)	0.0451 (7)	
N5	0.8682 (4)	0.6428 (2)	0.9347 (2)	0.0425 (7)	
N4	0.9624 (6)	0.7606 (3)	0.8611 (3)	0.0623 (10)	
N1	0.1431 (6)	0.5199 (3)	0.3912 (3)	0.0646 (10)	
N6	0.7523 (6)	0.5209 (2)	0.9947 (3)	0.0678 (12)	
N3	0.0650 (7)	0.7017 (3)	0.6196 (3)	0.0759 (13)	
C6	0.8422 (5)	0.5964 (2)	1.0067 (2)	0.0444 (8)	
C10	0.9484 (5)	0.7226 (2)	0.9377 (2)	0.0434 (8)	
C1	0.0856 (5)	0.5995 (3)	0.4122 (2)	0.0483 (9)	
C7	0.9051 (6)	0.6309 (3)	1.0881 (2)	0.0571 (11)	
C5	0.0425 (6)	0.6919 (2)	0.5325 (3)	0.0489 (9)	
C9	1.0121 (6)	0.7571 (3)	1.0189 (3)	0.0532 (10)	
C8	0.9889 (6)	0.7105 (3)	1.0922 (3)	0.0581 (11)	
C2	0.0069 (6)	0.6621 (4)	0.3543 (3)	0.0630 (12)	
C3	−0.0514 (6)	0.7378 (3)	0.3851 (3)	0.0651 (13)	
C4	−0.0359 (6)	0.7541 (3)	0.4729 (4)	0.0606 (12)	
H7	0.874 (6)	0.603 (3)	1.136 (3)	0.066 (13)*	
H2	0.000 (6)	0.648 (3)	0.298 (3)	0.074 (14)*	
H4	−0.066 (5)	0.803 (3)	0.498 (2)	0.047 (11)*	
H9	1.071 (6)	0.802 (3)	1.022 (3)	0.075 (15)*	
H8	1.035 (6)	0.735 (3)	1.146 (3)	0.062 (12)*	
H3	−0.108 (7)	0.784 (3)	0.345 (3)	0.094 (16)*	
H2N1	0.222 (6)	0.493 (3)	0.427 (3)	0.061 (14)*	
H2N4	1.020 (6)	0.808 (3)	0.863 (3)	0.067 (14)*	
H1N6	0.720 (7)	0.498 (3)	0.945 (3)	0.074 (15)*	
H1N2	0.157 (6)	0.581 (3)	0.530 (3)	0.058 (13)*	
H1N5	0.839 (6)	0.623 (3)	0.886 (3)	0.079 (16)*	
H1N3	0.026 (6)	0.748 (3)	0.645 (3)	0.062 (13)*	
H1N4	0.913 (6)	0.740 (3)	0.817 (3)	0.047 (13)*	
H2N6	0.747 (7)	0.488 (3)	1.036 (3)	0.082 (17)*	
H2N3	0.095 (9)	0.661 (4)	0.654 (4)	0.12 (2)*	
H1N1	0.139 (7)	0.508 (3)	0.331 (3)	0.089 (17)*	

### Atomic displacement parameters (Å^2^)

**Table:**

	*U*^11^	*U*^22^	*U*^33^	*U*^12^	*U*^13^	*U*^23^
Co1	0.0442 (3)	0.0358 (3)	0.0350 (2)	0.0012 (2)	0.00079 (19)	0.00112 (19)
Cl1	0.0558 (6)	0.0446 (5)	0.0472 (5)	−0.0080 (4)	0.0022 (4)	−0.0022 (4)
Cl2	0.0553 (6)	0.0531 (5)	0.0382 (5)	0.0072 (4)	−0.0049 (4)	−0.0042 (4)
Cl3	0.0573 (6)	0.0547 (6)	0.0547 (5)	0.0058 (5)	0.0170 (5)	0.0013 (5)
Cl4	0.0892 (8)	0.0407 (5)	0.0511 (6)	0.0053 (5)	−0.0141 (5)	0.0073 (4)
N2	0.0487 (19)	0.0386 (16)	0.0468 (17)	0.0056 (15)	0.0027 (15)	0.0068 (14)
N5	0.0458 (18)	0.0446 (17)	0.0360 (16)	−0.0034 (14)	0.0023 (13)	−0.0017 (14)
N4	0.068 (3)	0.059 (2)	0.059 (2)	−0.015 (2)	0.006 (2)	0.010 (2)
N1	0.061 (2)	0.077 (3)	0.054 (2)	0.011 (2)	0.0023 (19)	−0.011 (2)
N6	0.103 (3)	0.053 (2)	0.045 (2)	−0.028 (2)	0.001 (2)	−0.0006 (18)
N3	0.122 (4)	0.047 (2)	0.063 (3)	0.011 (2)	0.030 (3)	−0.001 (2)
C6	0.051 (2)	0.0416 (19)	0.0397 (19)	−0.0056 (17)	0.0037 (16)	−0.0002 (15)
C10	0.040 (2)	0.041 (2)	0.049 (2)	0.0030 (16)	0.0073 (16)	0.0048 (16)
C1	0.039 (2)	0.057 (2)	0.048 (2)	−0.0024 (18)	0.0032 (16)	0.0011 (18)
C7	0.078 (3)	0.057 (3)	0.035 (2)	−0.015 (2)	0.004 (2)	−0.0021 (18)
C5	0.054 (2)	0.041 (2)	0.053 (2)	0.0032 (18)	0.0151 (18)	0.0070 (18)
C9	0.050 (2)	0.046 (2)	0.062 (3)	−0.013 (2)	0.002 (2)	−0.0082 (19)
C8	0.070 (3)	0.058 (3)	0.044 (2)	−0.014 (2)	−0.002 (2)	−0.0083 (19)
C2	0.054 (3)	0.085 (3)	0.047 (2)	−0.006 (2)	0.000 (2)	0.016 (2)
C3	0.060 (3)	0.061 (3)	0.073 (3)	0.001 (2)	0.005 (2)	0.029 (2)
C4	0.059 (3)	0.040 (2)	0.085 (3)	0.009 (2)	0.019 (2)	0.015 (2)

### Geometric parameters (Å, º)

**Table:**

Co1—Cl3	2.2595 (14)	N6—H2N6	0.82 (5)
Co1—Cl4	2.2645 (11)	N3—C5	1.335 (6)
Co1—Cl2	2.2754 (11)	N3—H1N3	0.88 (4)
Co1—Cl1	2.2795 (13)	N3—H2N3	0.83 (6)
N2—C5	1.346 (5)	C6—C7	1.379 (5)
N2—C1	1.359 (5)	C10—C9	1.378 (5)
N2—H1N2	0.82 (4)	C1—C2	1.382 (6)
N5—C6	1.355 (4)	C7—C8	1.369 (6)
N5—C10	1.360 (5)	C7—H7	0.91 (4)
N5—H1N5	0.82 (5)	C5—C4	1.393 (6)
N4—C10	1.332 (5)	C9—C8	1.367 (6)
N4—H2N4	0.84 (5)	C9—H9	0.82 (5)
N4—H1N4	0.79 (4)	C8—H8	0.93 (4)
N1—C1	1.349 (5)	C2—C3	1.350 (7)
N1—H2N1	0.84 (5)	C2—H2	0.89 (4)
N1—H1N1	0.94 (5)	C3—C4	1.362 (7)
N6—C6	1.337 (5)	C3—H3	0.99 (5)
N6—H1N6	0.85 (5)	C4—H4	0.89 (4)
			
Cl3—Co1—Cl4	112.69 (5)	N4—C10—N5	117.1 (4)
Cl3—Co1—Cl2	109.64 (5)	N4—C10—C9	125.0 (4)
Cl4—Co1—Cl2	109.80 (4)	N5—C10—C9	117.9 (3)
Cl3—Co1—Cl1	110.73 (5)	N1—C1—N2	116.0 (4)
Cl4—Co1—Cl1	106.44 (5)	N1—C1—C2	126.5 (4)
Cl2—Co1—Cl1	107.37 (4)	N2—C1—C2	117.4 (4)
C5—N2—C1	124.2 (3)	C8—C7—C6	118.5 (4)
C5—N2—H1N2	123 (3)	C8—C7—H7	123 (3)
C1—N2—H1N2	113 (3)	C6—C7—H7	118 (3)
C6—N5—C10	124.1 (3)	N3—C5—N2	118.3 (4)
C6—N5—H1N5	120 (3)	N3—C5—C4	124.4 (4)
C10—N5—H1N5	116 (3)	N2—C5—C4	117.3 (4)
C10—N4—H2N4	117 (3)	C8—C9—C10	118.7 (4)
C10—N4—H1N4	120 (3)	C8—C9—H9	121 (3)
H2N4—N4—H1N4	123 (4)	C10—C9—H9	120 (3)
C1—N1—H2N1	119 (3)	C9—C8—C7	122.7 (4)
C1—N1—H1N1	117 (3)	C9—C8—H8	117 (3)
H2N1—N1—H1N1	118 (4)	C7—C8—H8	121 (3)
C6—N6—H1N6	124 (3)	C3—C2—C1	120.0 (4)
C6—N6—H2N6	121 (4)	C3—C2—H2	125 (3)
H1N6—N6—H2N6	114 (4)	C1—C2—H2	115 (3)
C5—N3—H1N3	122 (3)	C2—C3—C4	121.4 (4)
C5—N3—H2N3	123 (4)	C2—C3—H3	122 (3)
H1N3—N3—H2N3	114 (5)	C4—C3—H3	117 (3)
N6—C6—N5	118.1 (3)	C3—C4—C5	119.7 (4)
N6—C6—C7	123.7 (4)	C3—C4—H4	127 (2)
N5—C6—C7	118.1 (3)	C5—C4—H4	114 (3)

### Hydrogen-bond geometry (Å, º)

**Table:**

*D*—H···*A*	*D*—H	H···*A*	*D*···*A*	*D*—H···*A*
N1—H2*N*1···Cl2	0.84 (5)	2.69 (5)	3.432 (4)	147 (4)
N4—H2*N*4···Cl2^i^	0.84 (5)	2.65 (5)	3.465 (5)	162 (4)
N6—H1*N*6···Cl4	0.85 (5)	2.58 (5)	3.406 (4)	165 (4)
N2—H1*N*2···Cl2	0.82 (4)	2.44 (4)	3.240 (3)	168 (4)
N5—H1*N*5···Cl1	0.82 (5)	2.43 (5)	3.191 (3)	156 (4)
N3—H1*N*3···Cl4^ii^	0.88 (4)	2.53 (5)	3.390 (5)	166 (4)
N4—H1*N*4···Cl1	0.79 (4)	2.79 (4)	3.481 (5)	147 (4)
N6—H2*N*6···Cl3^iii^	0.82 (5)	2.75 (5)	3.516 (4)	156 (4)
N3—H2*N*3···Cl3	0.83 (6)	2.61 (6)	3.420 (5)	166 (6)
N1—H1*N*1···Cl1^iv^	0.94 (5)	2.69 (5)	3.326 (4)	126 (4)
C7—H7···Cl3^iii^	0.91 (4)	2.89 (4)	3.669 (4)	145 (3)

Symmetry codes: (i) −*x*+3/2, *y*+1/2, −*z*+3/2; (ii) −*x*+1/2, *y*+1/2, −*z*+3/2; (iii) −*x*+1, −*y*+1, −*z*+2; (iv) −*x*+1, −*y*+1, −*z*+1.
